# Quantitative Analysis in Combination with Fingerprint Technology and Chemometric Analysis Applied for Evaluating Six Species of Wild* Paris* Using UHPLC-UV-MS

**DOI:** 10.1155/2016/3182796

**Published:** 2016-12-21

**Authors:** Yuangui Yang, Ji Zhang, Hang Jin, Jinyu Zhang, Yuanzhong Wang

**Affiliations:** ^1^College of Traditional Chinese Medicine, Yunnan University of Traditional Chinese Medicine, Kunming 650500, China; ^2^Institute of Medicinal Plants, Yunnan Academy of Agricultural Sciences, Kunming 650200, China; ^3^Yunnan Technical Center for Quality of Chinese Materia Medica, Kunming 650200, China

## Abstract

A fast method was developed by ultra high performance liquid chromatography (UHPLC) for simultaneous determination of polyphyllin I and polyphyllin II. Chemometric analyses including principal component analysis (PCA) and partial least squares discriminant analysis (PLS-DA) based on UHPLC chromatography were used to evaluate 38 batches from six species of* Paris*. Variable importance of projection was applied to select important peaks. Meanwhile, similarity analysis of UHPLC fingerprint was used to evaluate the sample of* Paris polyphylla yunnanensis *(PPY) and* P. axialis* (PA). The results indicated that the total content of saponins in PPY and PA collected from Baoshan City of Yunnan Province above 8.07 mg/g was stronger than that from other areas of the rest of species. PLS-DA showed better performance than PCA with regard to classifying the samples. Retention time during 20–27 minutes of UHPLC was screened as significant peak for distinguishing* Paris* of different species and original geography. All of PPY and PA with similarity value were more than 0.80. It indicated that quantitative analysis combined with chemometric and similarity analyses could evaluate the different species of* Paris* effectively and comprehensively.

## 1. Introduction

As one of the oldest forms of health care, herbal medicine (HM) recorded by the World Health Organization (WHO) has been widely used for thousands of years due to undoubted prophylactic and therapeutic effects in the majority of the world' s region, particularly in developing countries [1]. It has been published by the United States Food and Drug Administration on the fundamentals of purity, safety, and efficacy [2]. Owing to complex chemical compositions, multiple analytical methodologies are used for assessment of HM.

Chromatographic fingerprint technologies can provide systematic and effective characteristics for quality control of HM [3]. They, therefore, have been introduced and accepted by the WHO [4]. Fingerprints combined with the chemometric analysis are widely applied to investigate the difference of chemical profile among the samples [5, 6]. Moreover, quantitative analysis can offer the content variation of the bioactive components clearly.

Rhizoma Paridis (RP) has been widely used for treatment of furunculosis, throat-swelling, bite wound, traumatic injury, and hyperspasmia for a long history. Steroid saponins, flavonoids, polysaccharoses, and aliphatic acids are the major bioactive components in RP [7]. Genus* Paris* (Family Liliaceae) has 22 species in China; up to now, only* Paris polyphylla* var.* yunnanensis* (PPY) and* P. polyphylla* var.* chinensis* (PPC) are recorded in Chinese Pharmacopoeia (CP) (2015 edition) as the significant materials of RP [8]. Other species, such as* P. cronquistii *(PC),* P. mairei *(PM),* P. fargesii *(PF), and* P. axialis *(PA) are regarded as RP to treat disease in the folk as well, especially in southwest of China.

As the main bioactive compounds, steroid saponins including polyphyllin I and polyphyllin II are recorded as the chimerical marker of CP. Modern pharmacology demonstrates that they have explicit effect on antitumor [9–11], antibacterial [12, 13], hemostatic [14, 15], anthelmintic [16, 17], and immuno-stimulating [18]* characteristics.* They are also used as the significant raw material of some Chinese patent drugs, such as “Biyan Qingdu Keli” and “Jidesheng Sheyao Tablet” [19]. A large amount of active constituents and RP saponins are insufficient due to the demand of domestic and international market [20]. Therefore, seeking and assessing the source of RP have become a task for the further study.

In previous investigations, several methods, for instance, high performance liquid chromatography (HPLC) [21–23], near infrared spectroscopy (NIR) [24], and ultraviolet spectroscopy (UV) [25], have been established for qualitative and quantitative analysis of the bioactive constituents of* Paris*. However, HPLC takes long analytical time and high consumption of solvent, and NIR needs the experienced technicians. In addition, UV requires numerous organic solvents and heavy workload.

Recently, ultrahigh performance liquid chromatography (UHPLC) with the fast and effective characteristic has been utilized to evaluate* Paris* qualitatively and quantitatively [26]. However, the drawback of this method is not comprehensive and reasonable for discrimination of different species and regions of* Paris*. Fortunately, UHPLC connected with the chemometric analysis can respond to the matter successfully. Meanwhile, fingerprint of UHPLC and chemometric analysis have been employed to evaluate the difference among the HM [27–29]. Therefore, the strategy which combined UHPLC technologies with chemometric could be used for quantitative evaluation and discrimination of* Paris*.

The aim of this study was to develop a rapid and sensitive UHPLC-UV-MS method for simultaneous determination of polyphyllin I and polyphyllin II in six species of 38 batches (Table 1). The reliable and synthetic quality control for PPY and PA was confirmed based on similarity analysis of chromatography. Additionally, according to the chromatographic fingerprint, chemometric analysis involved in principal component analysis (PCA) and partial least squares discriminant analysis (PLS-DA) were used to identify* Paris* of six species.

## 2. Experimental

### 2.1. Materials and Reagents

**Figure 1:**
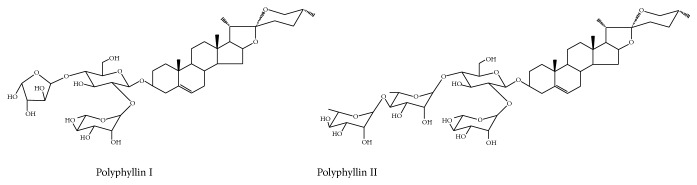
Structure of polyphyllin I and polyphyllin II.

A total of 38 batches of wild* Paris* were collected from Guizhou and Yunnan Provinces, southwestern China. The information of samples was listed in Table 1. The identification of* Paris* species was conducted by Professor Jinyu Zhang (Yunnan Academy of Agricultural Sciences). Voucher specimens were deposited in Institute of Medicinal Plants, Yunnan Academy of Agricultural Sciences. Fresh materials were washed by tap water, dried in sunshine, and ground into the powder screening 60 meshes. Chemical standards of polyphyllin I and polyphyllin II (Figure 1) were purchased from Chinese National Institute for the Control of Pharmaceutical and Biological Products (Beijing, China). The purity of all reference compounds was determined to be over 98% by UHPLC-UV-MS. Acetonitrile and methanol (chromatographic grade) were provided by Tedia Company (CT, USA). Formic acid (chromatographic grade) was purchased from Dikmapure Company (CA, USA). Deionized water was provided by a Milli-Q system (MA, USA). The other chemical reagents were of analytical grade.

### 2.2. Apparatus and Chromatographic Conditions

Chromatographic separation was conducted by a Shim-pack XR-ODS III column (150 × 2.0 mm, 2.2 *μ*m) using the Shimadzu LC-MS-8030 triple quadruple mass spectrometer (Kyoto, Japan) equipped with electrospray ionization (ESI) mode. The UHPLC system consisted of a degasser, binary gradient pumps, a column oven, and an autosampler. The chromatographic conditions included acetonitrile as the mobile phase A and 0.1% formic acid solution as the mobile phase B. The linear gradient elution was carried out as follows: 17% A, 0–1.5 min; 17–23% A, 1.5–4.0 min; 23% A, 4.0–8.7 min; 23–38% A, 8.7–18 min; 38–60% A, 18–25.6 min; 60–17% A, 25.6–28 min; the reequilibration time was 4 min and the total running was 32 min. The flow rate of mobile phase was 0.45 mL/min. The effluents from the column were detected by the UV detector where the detection wavelength was set at 203 nm, and the column temperature was 45°C. The mass spectrometer was performed as follows: the nebulizing gas was nitrogen with a flow rate 3.0 L/min; nitrogen was also used as drying gas at a flow rate 15.0 L/min. The capillary voltage was set up as 4.5 kV (ESI^−^). The temperature of desolvation and heat block were set at 250°C and 100°C, respectively. The MS data was acquired at a range from 100 to 1000 amu.

### 2.3. Preparation of Sample Solution and Reference Compounds Solution

For sample solution, sample powder was weighed 0.1 g accurately, transferring into 10 mL glass stopper tube and adding 2 mL of 80% methanol to dissolve the sample under sonication for 40 min at room temperature. The lost methanol was replenished. Then, the solution was filtered and the filtrate was screened a 0.22 *μ*m membrane filter. Each volume was injected into the UHPLC-UV-MS system with 1 *μ*L. Preparation of standard solutions: the appropriate amount of the standard components was dissolved 100% methanol and stored at 4°C before use. Then, the standard solution was prepared by stepwise dilution of corresponding stock solution with methanol/water (70 : 30, v/v) to appropriate concentration based on development of calibration curves.

### 2.4. Method Validation

The calibration curve for each reference standard was confirmed by plotting the peak area versus concentration. The Limits of detection (LOD) and quantification (LOQ) were accumulated by signal-to-noise of 3 and 10, respectively. The inter- and intraday were used to evaluate precision of the developed method and investigated by determining the two standards in six replicates a single day and three consecutive days. For the recovery, three different concentrations (50%, 100%, and 150% of the known amount in sample) were spiked into sample PPY2. The recovery rate was calculated by the formula [The recovery rate (%) = (measured amount − original amount)/amount added × 100%]. Stability of sample solution was investigated by peak areas of analytes after storing at room temperature for 0, 2, 4, 12, and 24 h. The inter-, intraday precision, recovery rate, and stability were expressed by relative standard deviation (RSD).

### 2.5. Data Analysis

Multivariate statistical analysis was used to classify the sample. To perform statistical analysis consisting of PCA and PLS-DA, the data of sample based on the UHPLC was imported into SIMCA-P^+^ 10.0 (Umetrics AB, Sweden). The samples of PPY and PA were carried out as the chromatographic condition, and the data analysis was performed by* Similarity Evaluation System for Chromatographic Fingerprint of Traditional Chinese Medicine* (Version 2004A), Chinese Pharmacopoeia Committee.

## 3. Results

### 3.1. Optimization of Extraction and UHPLC-UV-MS

**Figure 2:**
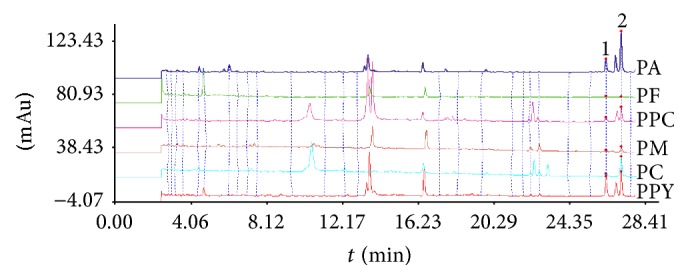
UHPLC chromatography of six species of* Paris*. Peaks 1 and 2 were responsible for polyphyllin II and polyphyllin I, respectively.

To obtain an effective extraction, the solvent (methanol, 80% methanol, and ethanol), methods (heating circumfluence and ultrasonic treatment), time (30, 40 and 50 min), and solid-liquid ratio (1 : 15, 1 : 20 and 1 : 25) were investigated. The results demonstrated that the optimum extracting condition was as follows: 80% methanol, ultrasonic treatment, and solid-liquid ratio 1 : 15 for 40 min at room temperature. Chromatographic conditions were optimized to achieve a satisfactory separation. Column size (150 × 2.0 mm, 2.2 *μ*m, and 75 × 2.0 mm, 1.6 *μ*m of the Shim-pack XR-ODS III), column temperature (35, 40, and 45°C), mobile phase (methanol: 0.05% formic acid solution, acetonitrile: 0.05% formic acid and acetonitrile: 0.1% formic acid), wavelength (203 and 210 nm), and flow rate (0.35, 0.4, and 0.45 mL/min) were investigated. The results showed that the optimum separation condition was as follows: column (150 × 2.0 mm, 2.2 *μ*m, and 45°C), mobile phase (acetonitrile: 0.05% formic acid) and the detection wavelength at 203 nm (according to the nature of steroid saponins), and the flow rate at 0.45 mL/min. In addition, the function of formic acid solution was not only to enhance the resolution, but also to restrain the ionization of saponins and to eliminate the peak tailing of target. Typical UHPLC chromatograms of* Paris* from six different species are shown in Figure 2.

### 3.2. Method Validation

The calibration curves for two standard constituents, LOD, LOQ, inter- and intraday are shown in Table 2. The relative standard deviations (RSD) of inter- and intraday are less than 2.98% and 2.81%. Stability of polyphyllin I and polyphyllin II was 3.46 and 2.78%, respectively. As shown in Table 3, the recovery rates of two polyphyllins range from 92.88% to 100.76%, and the RSD are less than 2.21%. The results indicated that the experiment was conducted accurately, precisely, and repeatedly.

### 3.3. Quantitative Analysis

**Figure 3:**
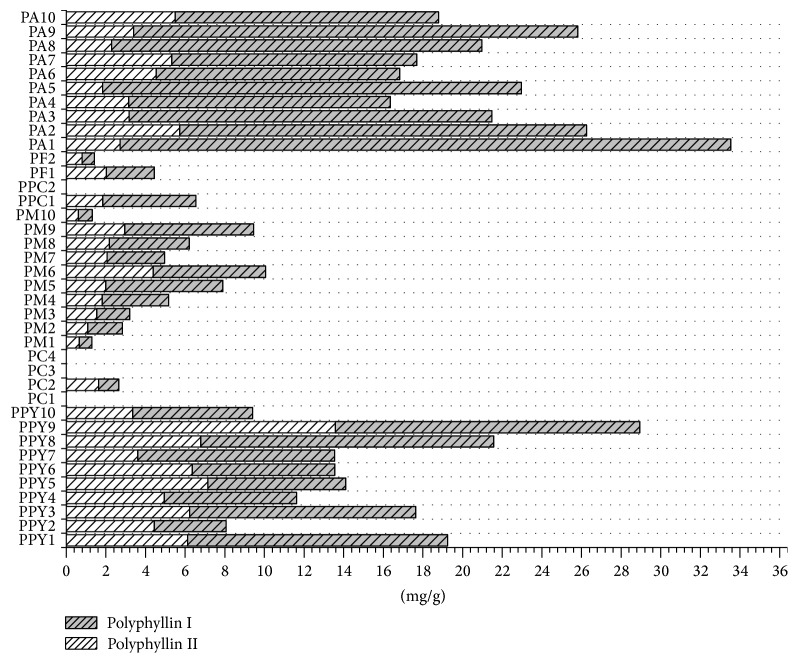
Content of polyphyllin I and polyphyllin II for six species of* Paris*.

Two standard components, namely, polyphyllin I and polyphyllin II, were authenticated by retention time, mass data, and the previous literature [22, 26]. The scans of polyphyllin I and polyphyllin II in the negative mode are shown in Supplementary Figure in Supplementary Material available online at http://dx.doi.org/10.1155/2016/3182796. The method was developed for simultaneous determination of two chemical markers including polyphyllin I and polyphyllin II in 38 batches of six species of* Paris*. The results are shown in Table 4 and Figure 3. Total saponins including polyphyllin I and polyphyllin II varied markedly among each sample from different regions and species. The total content of polyphyllin I and polyphyllin II in PA1 (33.53 mg/g) from Baoshan City of Yunnan Province was 25.8 times higher than that of PM 1 (1.30 mg/g) from Lijiang City of Yunnan Province. Moreover, it was hardly detected in the samples, namely, PC1 (Bijie, Guizhou), PC3 (Honghe, Yunnan), PC4 (Wenshan, Yunnan), and PPC1 (Zhaotong, Yunnan). The result indicated that the species variation may be an influence on the content of sample. Interestingly, for PPY and PA collected from Baoshan City of Yunnan Province, the total content above 8.07 mg/g was stronger than that from other areas of the rest of species, and the average content of PPY (15.77 mg/g) was lower than that of PA (22.07 mg/g). It could be found that the geographical origins may lead to different chemical constituents. It agreed with the previous investigation that geographical origins had a much greater influence on* Paris *[24].

Modern pharmacology displayed that these two bioactive markers were used for treatment disease of antitumor and gastroprotective agent [30, 31], but the mechanism of anticancer function might be different between them. Generally, polyphyllin I had a good performance on antileishmanial effect, while the effect of immune-stimulating activity was tested by polyphyllin II [32, 33]. In addition, integrated medicinal plants were usually utilized for protection and treatment of disease. The steroid saponins containing polyphyllin I and polyphyllin II were synergetic effects for RP. Therefore, the appropriate proportion of polyphyllin I and polyphyllin II might have a great influence on the therapeutic function of RP. As shown in Table 4 and Figure 3, the content of polyphyllin I was greatly higher than that of polyphyllin II with the ratio (equal to polyphyllin II against polyphyllin I) below 100% in the majority of samples; PA1 (8.78%) was the lowest ratio among the sample. On the contrary, five samples including PPY2 (122.44%), PPY5 (102.65%), PC2 (161.48%), PM1 (103.21%), and PF2 (127.69%) with regard to the content ratio were above 100%. In other words, the number of polyphyllin II was larger than polyphyllin I compared to other samples which had the various pharmacological functions. For PPY, the content ratio ranged from 46.61 to 122.44%, for PM (33.87–103.21%), PF (84.33–127.69%), and PA (8.61–43.08%). PPY had the apparent content ratio change while the moderate was for PA. The results indicated that the samples had the significant difference between polyphyllin II and polyphyllin I. The theory of traditional Chinese medicine is that multiconstituents were responsible for multitargets. Obviously, one or several markers or pharmacologically active components in herbs hardly tackle this urgent issue. It is therefore necessary to develop the synthetic, systematic, and effective method which includes chemometric and fingerprint analysis.

### 3.4. Principal Component Analysis

**Figure 4:**
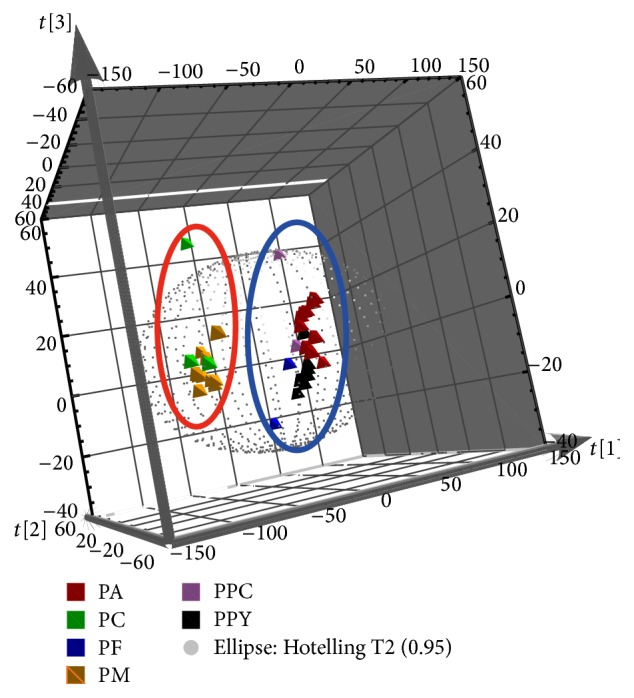
Three-dimensional score plot of principal component analysis for UHPLC fingerprint.

PCA, an unsupervised multivariate-data analytical method, concentrated the multidimensional information into a two- or three-dimensional data [34]. As one of the most significant multivariate analysis techniques, PCA was used to be responsible for the relationship between chemical information and samples [6]. In this study, PCA based on the UHPLC chromatography was performed by the chemical signal information ranging from 2.50 min to 27.99 min with 0.83 × 10^−2^ min of the interval time. Compared with the previous studies based on the common peak, this method included more bioactive chemical markers among the characteristic band. The aim of this study for PCA was used to distinguish the different species of* Paris* according to the data of UHPLC chromatography. As shown in Figure 4 and the three-dimensional score plot of PCA, samples were separated depending on the first three principal components (PC) 1, PC2, and PC3. The first three PC accounted for the 76.30% of the total variance to classify the sample. The results indicated that PF, PA, PPC, and PPY were clustered in positive PC1 score; by contrast, PC and PM were distributed in negative PC1 score. It was possible that four species of PF, PA, PPC, and PPY had similarly chemical information, especially sample of PA and PPY which had larger tight range. The results were similar to the quantitative analysis of PPY and PA. As official source of RP, the samples of PPC were not concentrated in one cluster. It was possible that geographical regions (PPC1 and PPC2 come from Wenshan and Zhaotong of Yunnan Province, resp.) had great impact on the chemical components. The PCA could separate the six species of* Paris* into two clusters well. However, it fails to explain the relationship among each species from different original geography. It is necessary to find an approach which could classify the sample well and select the significant peak.

### 3.5. Partial Least Squares Discriminant Analysis

**Figure 5:**
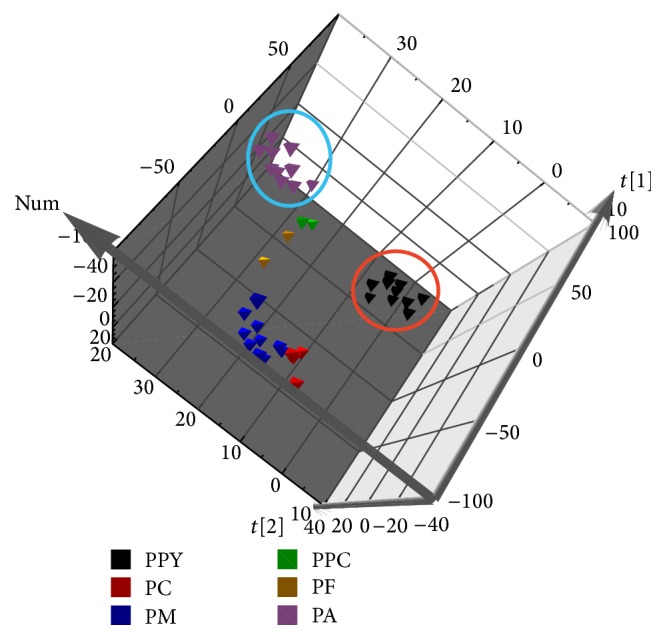
Three-dimensional score plot of partial least squares discriminant analysis for UHPLC fingerprint.

A supervised method, PLS-DA, is employed to identify signal feature because of serial groups confirmed according to the scores plot among groups [35]. For the* Paris*, a three-dimensional score scatter plot was built by UHPLC chromatography. As shown in Figure 5, the clustered species of* Paris* are in accordance with the result for PCA, namely, PPY, PPC, PF and PA in positive PC1, and PC and PM in negative PC1. Fortunately, the taxonomic species had a better performance consistent with PLS-DA compared to the strategy for PCA.

**Figure 6:**
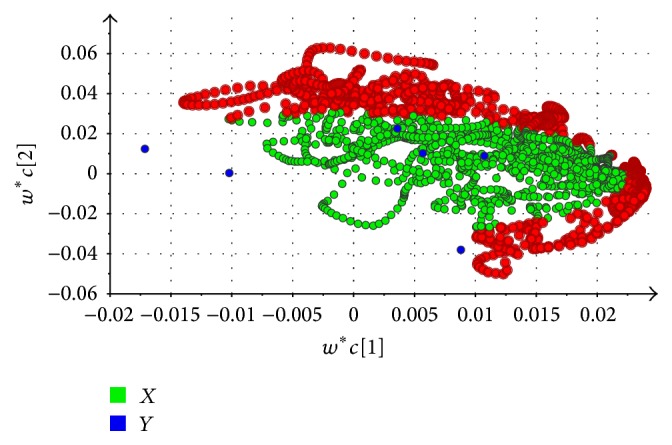
The loading scatter plots for UHPLC, “*X*” represents the information obtained by UHPLC chromatographic peaks and “*Y*” represents the six species of* Paris*. Marked spots represent the importance of composition for clustering that VIP above 1. The abscissa represents the weights for the first components and the ordinate represents the weights of the second components.

Variable importance of projection (VIP), as a variable selection approach, was employed for the further analysis of PLS-DA model [36]. A variable was treated as important to the PLS-DA model, when the VIP value > 1 [37]. It was therefore useful and simple strategy that VIP was used to find important variable [38]. As shown in Figure 6, the loading weights scatter plot displays the relation between the *X* variables and the *Y* variables. To perform this plot, VIP values above 1.0 were colored. A marked plot was responsible for a chromatographic signal. In this plot, 1757 intensity values with VIP above 1, 431 values (24.5%) were ranged from 2.5 to 10 minutes, 558 values (31.7%) were distributed from 10 to 20 minutes, and 766 values (43.5%) ranged from 20 to 27 minutes. It means that the peaks were regarded as significant to classify the species during retention time (*t*_*R*_) in 20–27 min. These significant values may include chromatographic peaks which are responsible for the steroid saponins whose polarity is higher than polyphyllin I (*t*_*R*_ = 26.9) according to the previous literature [26]. In addition, it can be seen that two bioactive chemical markers were above 1.0. The results agreed with quantitative analysis that polyphyllin I and polyphyllin II were treated as the chemical marker to differentiate between the different species and original geography for* Paris*. For six species of* Paris*, two species including PPY and PA had high content of steroid saponins in quantitative analysis and could be clustered reasonably by chemometric analysis. Moreover, each sample of PPY and PA and the content of two bioactive constituents could achieve the threshold (6 mg/g) in CP (2015 versions). To better investigate PPY and PA, UHPLC chromatographic fingerprint was employed.

### 3.6. Similarity Analysis of UHPLC Fingerprint of PPY and PA

**Figure 7:**
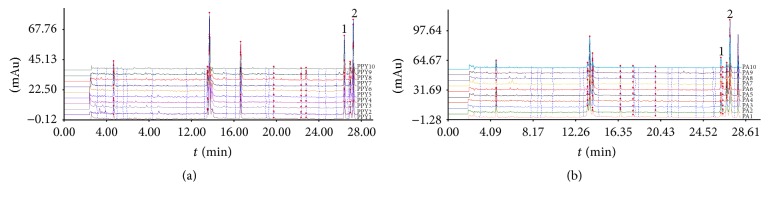
(a) UHPLC chromatograms fingerprint for PPY. (b) UHPLC chromatograms fingerprint for PA. Marked “1 and 2” are the represented polyphyllin II and polyphyllin I.

Similarity analysis, as a comprehensive and stable analysis technology, is used for quality control of HM. Under the chromatographic conditions, UHPLC chromatograms of PPY and PA were acquired. As shown in Figures 7(a)-7(b), it is indicated that the number of common peaks of PPY and PA were 32 and 25, respectively. These common peaks had bioactive components that we could notice beside polyphyllin I and polyphyllin II. The average chromatograms of ten batches of sample of PPY and PA were treated as the standard characteristic fingerprint of the PPY and PA, respectively. Similarity values were calculated by comparing each sample fingerprint pattern with the mean chromatogram. For all of samples, the similarity values of PPY and PA were above 0.80 and 0.82, respectively.

## 4. Discussion

In the quantitative analysis, the ten batches samples of PPY would be applied to clinic according to the current standard of CP (2015 edition) that the total content was above 6 mg/g [8], and the results were similar to the PCA and PLS-DA based on UHPLC fingerprint. A few markers and pharmacological active components were hardly used to evaluate quality control of sample. Chromatographic fingerprint, as a relatively comprehensive analysis approach, was accepted to evaluate a complex construction of HM. In this study, the similarity values were above 0.80 for samples of PPY and PA. It is demonstrated that similarity analysis for UHPLC could be developed as a tool to evaluate the quality and ensure the stability for PPY and PA. However, compared with other herbal, similarity values of sample were more than 0.90 [3, 11]. It indicated the chemical constituent varied among samples, the reason may be caused by habitat, processing, and storage different from others.

If one or several components were applied to pharmacy industry and clinic, it was suggested that numerous unofficial sources could be regarded as substitutes. For instance, the contents of polyphyllin I and polyphyllin II in PM were higher than that in PC and PF. These two constituents were extracted in PM instead of PPY or PPC. We could find that different geographical regions of PPC had diverse bioactive components. To the best of our knowledge, the quality of HM was corresponded with the habitat, harvesting time, and processing modes. For example, different geographical origin of green coffee (*Coffea arabica* and* Coffea canephora*) had different chemical partitioning and antioxidant capacity [39]. Influence of processing procedure on the quality of Radix Scrophulariae was investigated by using accelerated solvent extraction and high performance liquid chromatography [40]. It was a guideline for the further study.

In addition, the quality of PA, to some content, was better than that of PPY in quantitative and similarity analysis. Therefore, it is suggested that PA may be accepted by CP as an official source to meet resource shortage.

## 5. Conclusions

In the present study, a comprehensive and convenient approach was established for the identification and quality evaluation of* Paris*. The result indicated that quantitative analysis and chemometric and fingerprint analyses could provide an effective strategy to evaluate samples of PPY and PA. This analytical method exhibited some significant advantages: (I) 38 batches of samples in six species of* Paris* were investigated; (II) reducing analytical time and decreasing the organic solvent were studied when compared with the HPLC; (III) a fast method was built using UHPLC-UV-MS for simultaneous determination of polyphyllin I and polyphyllin II of different species in* Paris*; (IV) UHPLC chromatography technology combined with PCA and PLS-DA analysis were applied for identification and evaluation of* Paris* sample of different species; (V) VIP based on the PLS-DA was employed to search significant peaks; (VI) similarity analysis of UHPLC fingerprint was employed as a quality control technology of PPY and PA. In conclusion, a feasible and credible method which was used to not only control the quality of other HM, but also search for more source of RP was developed.

## Supplementary Material

 The scans of polyphyllin I and polyphyllin II in the negative mode.

## Figures and Tables

**Table 1:** Information of different species from different regions.

Number	Species	Site
PPY1-10	*Paris polyphylla* var. *yunnanensis*	Baoshan City of Yunnan Province
PC1-2	*Paris cronquistii*	Bijie City of Guizhou Province
PC3		Honghe City of Yunnan Province
PC4		Wenshan City of Yunnan Province
PM1-10	*Paris mairei*	Lijiang City of Yunnan Province
PPC1	*Paris polyphylla* var.* chinensis*	Wenshan City of Yunnan Province
PPC2		Zhaotong City of Yunnan Province
PA1-10	*Paris axialis*	Baoshan City of Yunnan Province
PF1	*Paris fargesii*	Bijie City of Guizhou Province
PF2		Bijie City of Guizhou Province

**Table 2:** Calibration curves, LOD, LOQ, intraday, and interday for polyphyllin I and polyphyllin II.

Analytes	Regression equation	*r* ^2^	LOD (*μ*g/mL)	LOQ (*μ*g/mL)	Interday (RSD, %)						Intraday (RSD, %)		Stability (RSD, %)
					Day 1		Day 2		Day 3				
					*t*	*p*	*t*	*p*	*t*	*p*	*t*	*p*	
Polyphyllin I	*Y* = 333.885*X* − 6750.93	0.9990	10.95	32.08	0.15	2.72	0.07	1.36	0.06	2.01	0.12	2.48	3.46
Polyphyllin II	*Y* = 271.889*X* − 5243.32	0.9997	14.67	44.44	0.16	2.88	0.06	2.98	0.05	2.11	0.12	2.81	2.78

**Table 3:** Recovery rate of polyphyllin I and polyphyllin II.

	Original amount (mg/g)	Amount added (mg/g)	Measured amount (mg/g)	Recovery rate (%)	RSD (%)
Polyphyllin I	3.62	1.75	5.31	98.57	1.57
		3.50	7.13	100.30	
		5.25	8.91	100.76	

Polyphyllin II	4.44	2.25	6.53	92.88	2.21
		4.50	8.94	100.00	
		6.75	11.03	97.63	

**Table 4:** Content of polyphyllin I and polyphyllin II of six species of 38 batches.

	Polyphyllin I (mg/g)	Polyphyllin II (mg/g)	Total saponins (mg/g)	Polyphyllin II/polyphyllin I (%)
PPY1	13.13	6.12	19.25	46.61
PPY2	3.63	4.44	8.07	122.44
PPY3	11.41	6.22	17.63	54.55
PPY4	6.68	4.95	11.62	74.06
PPY5	6.96	7.15	14.11	102.65
PPY6	7.20	6.36	13.56	88.30
PPY7	9.94	3.60	13.54	36.23
PPY8	14.79	6.78	21.57	45.86
PPY9	15.36	13.58	28.94	88.47
PPY10	6.05	3.35	9.40	55.31
PC1	—	—	—	—
PC2	1.01	1.63	2.64	161.48
PC3	—	—	—	—
PC4	—	—	—	—
PM1	0.64	0.66	1.30	103.21
PM2	1.75	1.07	2.83	61.17
PM3	1.65	1.54	3.19	93.07
PM4	3.35	1.81	5.16	54.12
PM5	5.90	2.00	7.90	33.87
PM6	5.66	4.39	10.05	77.51
PM7	2.90	2.06	4.96	70.98
PM8	4.02	2.18	6.20	54.22
PM9	6.49	2.96	9.44	45.56
PM10	0.70	0.61	1.30	87.60
PPC1	4.69	1.84	6.54	39.35
PPC2	—	—	—	—
PF1	2.41	2.03	4.44	84.33
PF2	0.62	0.80	1.42	127.69
PA1	30.83	2.71	33.53	8.78
PA2	20.53	5.73	26.26	27.89
PA3	18.31	3.16	21.47	17.25
PA4	13.20	3.14	16.35	23.81
PA5	21.15	1.82	22.98	8.61
PA6	12.29	4.53	16.82	36.90
PA7	12.37	5.33	17.69	43.08
PA8	18.69	2.29	20.98	12.25
PA9	22.42	3.39	25.81	15.10
PA10	13.29	5.50	18.79	41.35

“—” Compounds were not detected.
