# Supplementary material for: Quantitative Analysis in Combination with Fingerprint Technology and Chemometric Analysis Applied for Evaluating Six Species of Wild Paris Using UHPLC-UV-MS
Source: J Anal Methods Chem. 2016 Dec 21;2016:3182796. doi: 10.1155/2016/3182796 (PMC5209620; doi:10.1155/2016/3182796)
Supplement: Supplementary file 1 — The scans of polyphyllin I and polyphyllin II in the negative mode. [file 3182796.f1.pdf]

Figure supplement

a

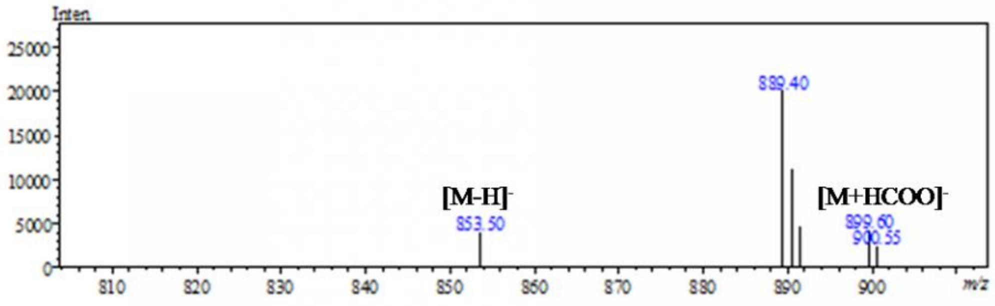

b

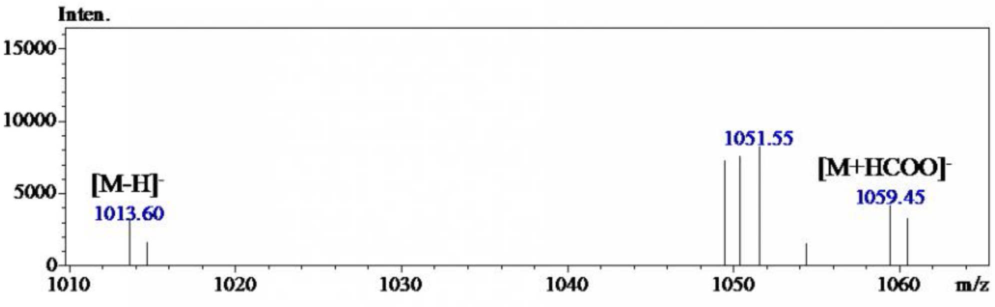

Fig. supplement a and b was responsible for the scan of polyphyllin I and polyphyllin II in the negative mode.
